# Trends in resource utilization for new‐onset psychosis hospitalizations at children's hospitals

**DOI:** 10.1002/jhm.13597

**Published:** 2025-02-02

**Authors:** Ankita Gupta, Matt Hall, Benjamin Masserano, Averi Wilson, Katherine Johnson, Clifford Chen, Lasya Challa, Harita Katragadda, Vineeta Mittal

**Affiliations:** ^1^ Department of Pediatrics, Division of Pediatric Hospital Medicine University of Texas Southwestern Dallas Texas USA; ^2^ Children's Medical Center Dallas Texas USA; ^3^ Children's Hospital Association Lenexa Kansas USA

## Abstract

**Background:**

Children with new‐onset psychosis often require hospitalization for medical evaluation.

**Objectives:**

The goal of this study was to assess variations in the management of children with new‐onset psychosis and characterize trends in resource utilization.

**Methods:**

The study included index hospitalizations for children ages 7–18 admitted to children's hospitals with a primary diagnosis of psychosis from 2011 to 2022 using the Pediatric Health Information System (PHIS) database. Children with a complex chronic condition were excluded. Resource utilization categories included medication, imaging, laboratory, and other clinical resources. Variability in resource utilization was assessed using covariance tests for random intercepts with generalized linear modes after adjusting for age, sex, payor, and severity. Trends in resource utilization were examined using generalized estimating equations adjusting for the same factors and accounting for hospital clustering.

**Results:**

Our data set included 7126 new‐onset psychosis hospitalizations from 37 children's hospitals. Teenage males and non‐Hispanic Whites were most likely to be hospitalized. There was a significant variation in resource utilization across hospitals in all categories (*p* < .001). The most frequently utilized resources were antipsychotic medications (76%), serum chemistry (77%), toxicology labs (72%), and brain magnetic resonance imaging (22%). The most notable increases in utilization were in the performance of laboratory tests, brain imaging, anesthetic use, and intravenous immunoglobulin use.

**Conclusion:**

Study findings suggest that there has been a stable rate of hospitalization for children with new‐onset psychosis, yet a significant variation in the medical evaluation exists. Significant increases and variations in resource utilization across all categories suggest an emerging need for robust evidence and consensus‐based practice guidelines.

## INTRODUCTION

Psychosis, defined as impaired reality with disruptions in thinking, perceptions, and behavior^1^, is one of the most frequent and costly pediatric mental health diagnoses requiring hospitalization.^2^ Symptoms can include hallucinations, delusions, disorganized speech, and other abnormal psychomotor behaviors.^3^ Each year, 100,000 young adults experience new‐onset psychosis in the United States, with peak onset between ages 15 and 25.^1^, ^4^ Recent data suggest that the prevalence of psychosis symptoms in pediatric patients is approximately 17% for children between 9 and 12 years of age and 7.5% among children between 13 and 18 years of age.^5^

Despite its incidence, managing psychosis in an inpatient setting remains a clinical challenge. Most patients with new‐onset psychosis do not go on to develop a primary psychotic disorder, and the differential diagnosis for secondary psychosis is broad.^1^, ^6^ Secondary psychosis can be the presenting feature of multiple medical conditions such as neurologic disorders, endocrine disorders, drug or toxin‐mediated syndromes, and others.^7^ Expert opinions vary on the extent of medical evaluation that patients should undergo during an index hospitalization.^8^, ^9^, ^10^ Crucially, a delay in or nontreatment of secondary psychosis in pediatric inpatients can lead to significant morbidity and even mortality.^7^

Not enough is known about how to evaluate a child presenting with new‐onset psychosis in the hospital setting. Thus, the objectives of this study were to assess variations in the evaluation and management of children hospitalized with a new‐onset psychosis across US children's hospitals and to characterize trends in resource utilization over time.

## METHODS

This was a retrospective, cross‐sectional study of the Pediatric Health Information System (PHIS; Children's Hospital Association) database, which contains data from 49 tertiary care children's hospitals. Of the 49 hospitals, 37 consistently participated in PHIS during the study period and were included in the study. All submitted data are de‐identified. Data quality and reliability are assured through a joint effort between the Children's Hospital Association and the respective hospitals. This study was approved for exemption by the institutional review board of the University of Texas Southwestern.

### Study population

Children 7–18 years of age with a primary discharge diagnosis of psychosis between January 2011 and December 2022 were included. Patient‐level variables included age (stratified as 7–10, 11–14, and 15–18), gender, race and ethnicity, payor (government, private, or other), discharge disposition (home, skilled facility, or other), and year (stratified as 2011–2013, 2014–2016, 2017–‐2019, and 2020–2022). Only index encounters were included to capture new‐onset psychosis. Children with complex chronic conditions were excluded as they may have underlying conditions affecting their evaluation and management.^11^ The Childhood and Adolescent Mental Health Disorders Classification System (CAMHD‐CS) was used to identify diagnoses that met the criteria for inclusion.^12^ Diagnoses were manually identified under categories of bipolar disorders, schizophrenia, and other psychotic disorders to best define a patient with psychosis symptoms (Appendix A).

### Measures of resource utilization

Utilization was identified from billing data and categorized as medication, imaging, laboratory, and other clinical resources^10^ (Appendix B). Medications included anesthetics, antidepressants, antiemetics, antiepileptics, antipsychotics, anxiolytics, intravenous immunoglobulin (IVIG), mood stabilizers, and steroids. Imaging included brain magnetic resonance imaging ((MRI), head computer tomography (CT), head ultrasound, abdominal imaging, reproductive imaging, and spinal imaging. Laboratory studies evaluated blood, urine, and cerebrospinal fluid samples and were categorized as autoimmune, chemistry, cerebrospinal (CSF) studies, endocrine, thyroid labs, hematology, infectious disease, toxicology, and urine studies. Other clinical resources included electroencephalogram (EEG), electrocardiogram (EKG), and therapy services such as physical (PT), occupational (OT), and speech therapy (ST). The tests and treatments were included based on existing literature, our local experience, availability at most children's hospitals, and data from the PHIS database.

### Statistical analysis

Categorical variables were summarized using frequencies and percentages. Variability in resource use across hospitals was assessed using covariance tests for random intercepts with generalized linear models with binomial distributions after adjusting for age, sex, payor, and severity. Trends in resource utilization across years were examined using generalized estimating equations adjusting for age, sex, payor, severity, and accounting for hospital clustering. All statistical analyses were performed using SAS version 9.4 (SAS Institute), and *p* < .05 was considered statistically significant.

## RESULTS

This study included 7126 new‐onset psychosis hospitalizations from 37 hospitals. About half of them were male and had government payors, and a large proportion (80.6%) were discharged home following hospitalization (Table 1).

**Table 1:** Demographic and clinical characteristics of children hospitalized with new‐onset psychosis.

		*N* (%)
Age (years)	7–10	883 (12.4)
	11–14	2080 (29.2)
	15–18	4163 (58.4)
Gender	Male	4138 (58.1)
	Female	2986 (41.9)
Race and ethnicity	Non‐Hispanic White	2958 (41.5)
	Non‐Hispanic Black	2129 (29.9)
	Hispanic	1256 (17.6)
	Asian	162 (2.3)
	Other	621 (8.7)
Payor	Government	3716 (57.5)
	Private	2346 (36.3)
	Other	401 (6.2)
Discharge disposition	Home	5215 (80.7)
	Skilled Facility	132 (1.9)
	Other^a^	1116 (17.3)
Year	2011–2013	1704 (23.9)
	2014–2016	1863 (26.1)
	2017–2019	1674 (23.5)
	2020–2022	1885 (26.5)

^a^ Other Discharge Disposition includes transfer to a Psychiatric Hospital (67.8%) Designated Cancer Center or Children's Hospital (11.2%), Type of Health Care Institution not Defined Elsewhere (6.3%), Against Medical Advice (4.2%), and Inpatient Rehabilitation Facility (1.8%).

### Variations in resource utilization

**Figure 1:**
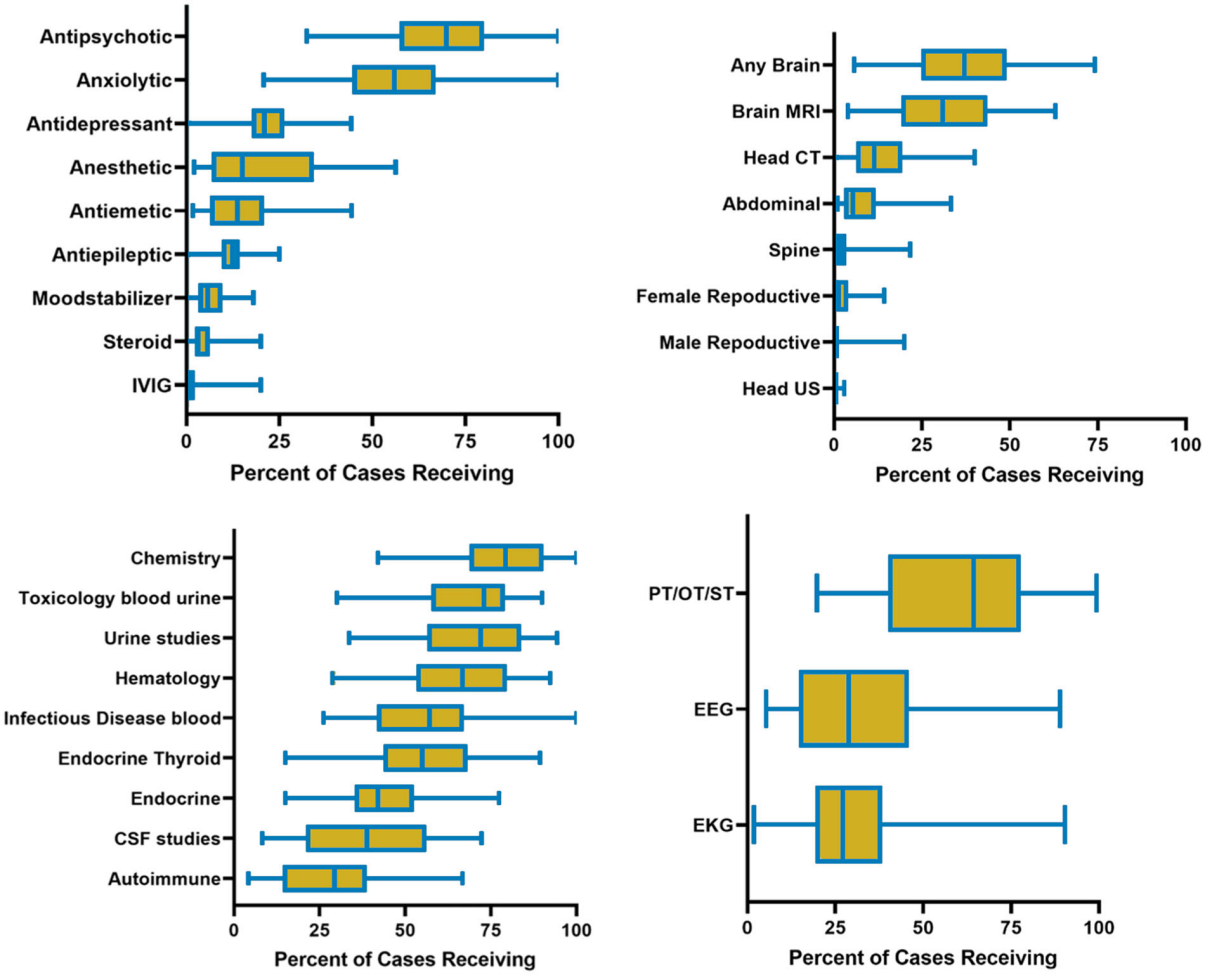
Variability across hospitals in resource utilization.

There was a statistically significant variation in resource use across all hospitals. This variation was significant in all categories of medications, imaging, laboratory, and other clinical resources (all *p* < .01, Figure 1). The most notable variation was in the utilization of CSF studies (median: 38.8 [interquartile range (IQR): 21.8, 55.8]), brain MRI (median: 30.9, [IQR: 19.3, 42.9]), anesthetic use (median: 15, [IQR: 7.6–33.3]), and use of therapy services (median: 64.3, [IQR: 43.8,75.4]).

### Trends in resource utilization

**Figure 2:**
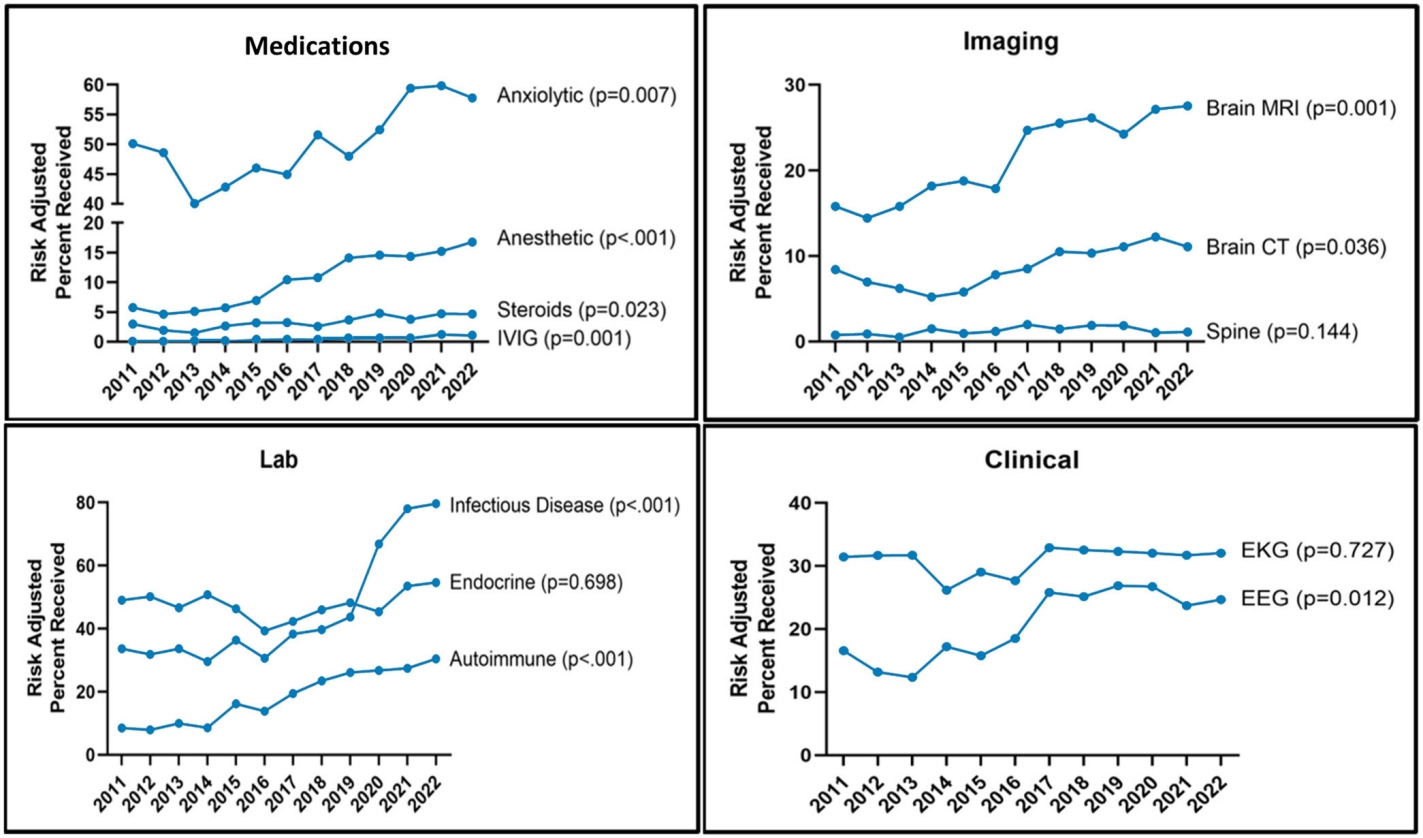
Risk adjusted* trends in select resources across years. *Risk adjusted for age, sex, payor, severity, and accounting for hospital clustering. CT, computed tomography; IVIG, immunoglobulin; MRI, magnetic resonance imaging.

Although the number of children hospitalized with new‐onset psychosis did not increase notably over the study period (*p* = .059; Table 1), resource utilization has significantly increased in all categories including medications, imaging, laboratory, and other clinical resources (all *p* < .001, Figure 2).

(a) *Medication utilization:* Trends suggest a significant increase in the use of anesthetic medication (adjusted odds ratio [aOR]: 3.4, 95% confidence interval [CI]: 2.0–5.8) and IVIG (aOR: 1.7, 95% CI: 1.3–2.2) (Figure 2).
(b) *Imaging utilization*: Brain MRI use (aOR: 1.9, 95% CI: 1.4–2.5) has significantly increased over time. Head CT was commonly performed, and frequency was stable over the course of the study.
(c) *Laboratory utilization*: Trends in laboratory utilization suggest a significant increase in the use of autoimmune labs (aOR: 4.1, 95% CI: 2.5–6.7) and infectious disease labs (aOR: 6.2, 95% CI: 3.7–10.4).
(d) *Other clinical resources*: Performance of both EEG (aOR: 2.1, 95% CI: 1.4–3.1) and EKGs (aOR: 1.0, 95% CI: 0.6–1.6) have increased over time (*p* = .012 and *p* = .72, respectively).

## DISCUSSION

Our multicenter, retrospective study results show that there is a significant variation across children's hospitals in the management of children with new‐onset psychosis. Findings also suggest an increase in resource utilization over the study period.

There is significant interhospital variability in the management of patients with new‐onset psychosis across all four resource categories, most notable in CSF studies, brain MRI, anesthetic use, and use of therapy services. This variation in the management may be due to a number of reasons. Many medical conditions such as infectious disease, endocrinopathies, drug and toxin ingestions, and neurological conditions can present with symptoms of new‐onset psychosis.^10^, ^13^ Because psychosis is often the only presenting symptom, the broad differential can make it challenging to limit the labs and imaging studies that are ordered for an otherwise undifferentiated patient, even after a thorough history and physical exam.^9^, ^14^ Variability may also exist because of a lack of national, standardized guidelines for diagnostic approaches to this patient population.^15^ National guidelines can facilitate a stepwise approach to the identification and management of common and uncommon diagnoses.^16^ Balancing early, accurate diagnosis with judicious use of healthcare resources for these patients is important. Nondiagnostic or low‐yield testing may delay diagnosis and treatment of psychosis,^17^, ^18^, ^19^, ^20^ and misdiagnosis or unnecessary treatment can be equally harmful.^4^ Our findings add to existing literature that highlights the need for future multidisciplinary investigation to help optimize the care of this complex patient population.

There are several implications when attempting to understand the increase in utilization of resources from 2011 to 2022, despite a stable rate of hospitalization for new‐onset psychosis. The increase in diagnostic testing for new‐onset psychosis may be part of a global trend of increasing unnecessary testing across the entire healthcare spectrum.^21^ As previously mentioned, the lack of consensus guidelines can place physicians caring for children with new‐onset acute psychosis in a difficult situation when deciding the extent of medical workup to pursue. Part of the reason for increased resource utilization could be related to increased awareness of and/or prevalence of pediatric mental health disorders in the last decade.^2^, ^22^, ^23^ The increase in utilization of anesthetic medication, brain MRI, and CSF studies is of particular clinical interest. A potential explanation may be due to an increased concern for neurologic causes of new‐onset psychosis such as autoimmune encephalitis (AIE).^24^, ^25^ In 2014, psychosis was found to be the 3rd most resource‐demanding pediatric inpatient mental health diagnosis.^2^ While our study did not specifically analyze costs related to resource utilization for children with new‐onset psychosis, it does shed light on the potential impact of increasing imaging, labs, and other testing over time. Increased utilization of laboratory tests such as infectious disease labs, hematology labs, and autoimmune labs may be of little consequence as the benefits of lab work far outweigh the risks. However, increased utilization of invasive procedures such as brain MRI and lumbar puncture to obtain CSF studies, which often require sedation in the pediatric population, must be approached in a thoughtful and considerate manner. This study did not allow an analysis of any results or outcomes of medical testing and evaluation that was conducted. However, Muhrer et al. suggested that extensive medical workup for new‐onset psychosis is typically unrevealing.^26^ This is similar to prior studies done in the adult patient population.^27^ For example, structural brain imaging in children with new‐onset psychosis is often unrevealing and does not affect the management of patients undergoing medical evaluation.^28^, ^29^, ^30^ Since caring for children often involves multiple disciplines, perhaps a strategy to manage resources and mitigate risks could be to approach care in a multidisciplinary fashion with hospitalists, neurologists, and psychiatrists sharing clinical perspectives regarding the extent of medical testing that should be done in each case.^15^

There are several limitations to this study. First, the study population was identified using primary discharge diagnosis codes, which are subject to coding errors. A lack of an established definition for “new‐onset psychosis” and the heterogeneity of the population make it possible that the 10th revision of the International Classification of Diseases (ICD‐10) codes selected in this study may not be fully representative of the population. This introduces the opportunity for misclassification bias in our analysis. Also, the details of the initial presentation could not be captured. An understanding of the patient's initial presentation including the presence or absence of focal neurologic findings on exam or any “red flag” features on history would provide more insight into the trends in resource utilization. The inability to capture the full clinical picture could lead to interpretation bias. Additionally, the PHIS database uses billing data to identify resource utilization, but it cannot capture the indication for an intervention or the results of any test. Patients receiving intense psychotic therapies may need tests and treatments for monitoring and titration of medicines including sedation during testing. Thus, it is possible that the study findings, specifically regarding resource utilization over time, are overstated or lack important clinical context. Lastly, this study is limited to practices at tertiary care children's hospitals and cannot be generalized to all settings that may care for children with new‐onset psychosis.

## CONCLUSIONS

In conclusion, our study suggests that there is a significant variation across children's hospitals in the evaluation and management of children hospitalized with new‐onset psychosis. Resource utilization for the initial workup for this patient population increased significantly in all examined categories. The next steps would involve additional subgroup analyses within conditions presenting as acute psychosis. This could then help develop an evidence‐based practice guideline to guide clinicians in managing hospitalized children with new‐onset psychosis.

## CONFLICT OF INTEREST STATEMENT

The authors declare no conflicts of interest.

## APPENDIX A

**Table:**

Mental health disorder group	International classification of diseases code	Version	Description
Bipolar and related disorders	2967	9	Bipolar I disorder, most recent episode (or current) unspecified
Bipolar and related disorders	29600	9	Bipolar I disorder, single manic episode, unspecified
Bipolar and related disorder	29601	9	Bipolar I disorder, single manic episode, mild
Bipolar and related disorders	29602	9	Bipolar I disorder, single manic episode, moderate
Bipolar and related disorders	29604	9	Bipolar I disorder, single manic episode, severe, specified as with psychotic behavior
Bipolar and related disorders	29644	9	Bipolar I disorder, most recent episode (or current) manic, severe, specified as with psychotic behavior
Bipolar and related disorders	29654	9	Bipolar I disorder, most recent episode (or current) depressed, severe, specified as with psychotic behavior
Bipolar and related disorders	F302	10	Manic episode, severe with psychotic symptoms
Mental health symptom	7801	9	Hallucinations
Mental health symptom	R440	10	Auditory hallucinations
Mental health symptom	R441	10	Visual hallucinations
Mental health symptom	R442	10	Other hallucinations
Mental health symptom	R443	10	Hallucinations, unspecified
Mental health symptom	R462	10	Strange and inexplicable behavior
Miscellaneous	F28	10	Other psychotic disorders not due to a substance or known physiological condition
Neurocognitive disorders	29381	9	Psychotic disorder with delusions in conditions classified elsewhere
Neurocognitive disorders	29382	9	Psychotic disorder with hallucinations in conditions classified elsewhere
Schizophrenia spectrum and other psychotic disorders	2970	9	Paranoid state, simple
Schizophrenia spectrum and other psychotic disorders	2971	9	Delusional disorder
Schizophrenia spectrum and other psychotic disorders	2972	9	Paraphrenia
Schizophrenia spectrum and other psychotic disorders	2973	9	Shared psychotic disorder
Schizophrenia spectrum and other psychotic disorders	2978	9	Other specified paranoid states
Schizophrenia spectrum and other psychotic disorders	2979	9	Unspecified paranoid state
Schizophrenia spectrum and other psychotic disorders	2980	9	Depressive type psychosis
Schizophrenia spectrum and other psychotic disorders	2981	9	Excitative type psychosis
Schizophrenia spectrum and other psychotic disorders	2983	9	Acute paranoid reaction
Schizophrenia spectrum and other psychotic disorders	2984	9	Psychogenic paranoid psychosis
Schizophrenia spectrum and other psychotic disorders	2988	9	Other and unspecified reactive psychosis
Schizophrenia spectrum and other psychotic disorders	2989	9	Unspecified psychosis
Schizophrenia spectrum and other psychotic disorders	29500	9	Simple type schizophrenia, unspecified
Schizophrenia spectrum and other psychotic disorders	29501	9	Simple type schizophrenia, subchronic
Schizophrenia spectrum and other psychotic disorders	29502	9	Simple type schizophrenia, chronic
Schizophrenia spectrum and other psychotic disorders	29503	9	Simple type schizophrenia, subchronic with acute exacerbation
Schizophrenia spectrum and other psychotic disorders	29504	9	Simple type schizophrenia, chronic with acute exacerbation
Schizophrenia spectrum and other psychotic disorders	29505	9	Simple type schizophrenia, in remission
Schizophrenia spectrum and other psychotic disorders	29510	9	Disorganized type schizophrenia, unspecified
Schizophrenia spectrum and other psychotic disorders	29511	9	Disorganized type schizophrenia, subchronic
Schizophrenia spectrum and other psychotic disorders	29512	9	Disorganized type schizophrenia, chronic
Schizophrenia spectrum and other psychotic disorders	29513	9	Disorganized type schizophrenia, subchronic with acute exacerbation
Schizophrenia spectrum and other psychotic disorders	29514	9	Disorganized type schizophrenia, chronic with acute exacerbation
Schizophrenia spectrum and other psychotic disorders	29515	9	Disorganized type schizophrenia, in remission
Schizophrenia spectrum and other psychotic disorders	29520	9	Catatonic type schizophrenia, unspecified
Schizophrenia spectrum and other psychotic disorders	29521	9	Catatonic type schizophrenia, subchronic
Schizophrenia spectrum and other psychotic disorders	29522	9	Catatonic type schizophrenia, chronic
Schizophrenia spectrum and other psychotic disorders	29523	9	Catatonic type schizophrenia, subchronic with acute exacerbation
Schizophrenia spectrum and other psychotic disorders	29524	9	Catatonic type schizophrenia, chronic with acute exacerbation
Schizophrenia spectrum and other psychotic disorders	29525	9	Catatonic type schizophrenia, in remission
Schizophrenia spectrum and other psychotic disorders	29530	9	Paranoid type schizophrenia, unspecified
Schizophrenia spectrum and other psychotic disorders	29531	9	Paranoid type schizophrenia, subchronic
Schizophrenia spectrum and other psychotic disorders	29532	9	Paranoid type schizophrenia, chronic
Schizophrenia spectrum and other psychotic disorders	29533	9	Paranoid type schizophrenia, subchronic with acute exacerbation
Schizophrenia spectrum and other psychotic disorders	29534	9	Paranoid type schizophrenia, chronic with acute exacerbation
Schizophrenia spectrum and other psychotic disorders	29535	9	Paranoid type schizophrenia, in remission
Schizophrenia spectrum and other psychotic disorders	29540	9	Schizophreniform disorder, unspecified
Schizophrenia spectrum and other psychotic disorders	29541	9	Schizophreniform disorder, subchronic
Schizophrenia spectrum and other psychotic disorders	29542	9	Schizophreniform disorder, chronic
Schizophrenia spectrum and other psychotic disorders	29543	9	Schizophreniform disorder, subchronic with acute exacerbation
Schizophrenia spectrum and other psychotic disorders	29544	9	Schizophreniform disorder, chronic with acute exacerbation
Schizophrenia spectrum and other psychotic disorders	29545	9	Schizophreniform disorder, in remission
Schizophrenia spectrum and other psychotic disorders	29550	9	Latent schizophrenia, unspecified
Schizophrenia spectrum and other psychotic disorders	29551	9	Latent schizophrenia, subchronic
Schizophrenia spectrum and other psychotic disorders	29552	9	Latent schizophrenia, chronic
Schizophrenia spectrum and other psychotic disorders	29553	9	Latent schizophrenia, subchronic with acute exacerbation
Schizophrenia spectrum and other psychotic disorders	29554	9	Latent schizophrenia, chronic with acute exacerbation
Schizophrenia spectrum and other psychotic disorders	29555	9	Latent schizophrenia, in remission
Schizophrenia spectrum and other psychotic disorders	29560	9	Schizophrenic disorders, residual type, unspecified
Schizophrenia spectrum and other psychotic disorders	29561	9	Schizophrenic disorders, residual type, subchronic
Schizophrenia spectrum and other psychotic disorders	29562	9	Schizophrenic disorders, residual type, chronic
Schizophrenia spectrum and other psychotic disorders	29563	9	Schizophrenic disorders, residual type, subchronic with acute exacerbation
Schizophrenia spectrum and other psychotic disorders	29564	9	Schizophrenic disorders, residual type, chronic with acute exacerbation
Schizophrenia spectrum and other psychotic disorders	29565	9	Schizophrenic disorders, residual type, in remission
Schizophrenia spectrum and other psychotic disorders	29570	9	Schizoaffective disorder, unspecified
Schizophrenia spectrum and other psychotic disorders	29571	9	Schizoaffective disorder, subchronic
Schizophrenia spectrum and other psychotic disorders	29572	9	Schizoaffective disorder, chronic
Schizophrenia spectrum and other psychotic disorders	29573	9	Schizoaffective disorder, subchronic with acute exacerbation
Schizophrenia spectrum and other psychotic disorders	29574	9	Schizoaffective disorder, chronic with acute exacerbation
Schizophrenia spectrum and other psychotic disorders	29575	9	Schizoaffective disorder, in remission
Schizophrenia spectrum and other psychotic disorders	29580	9	Other specified types of schizophrenia, unspecified
Schizophrenia spectrum and other psychotic disorders	29581	9	Other specified types of schizophrenia, subchronic
Schizophrenia spectrum and other psychotic disorders	29582	9	Other specified types of schizophrenia, chronic
Schizophrenia spectrum and other psychotic disorders	29583	9	Other specified types of schizophrenia, subchronic with acute exacerbation
Schizophrenia spectrum and other psychotic disorders	29584	9	Other specified types of schizophrenia, chronic with acute exacerbation
Schizophrenia spectrum and other psychotic disorders	29585	9	Other specified types of schizophrenia, in remission
Schizophrenia spectrum and other psychotic disorders	29590	9	Unspecified schizophrenia, unspecified
Schizophrenia spectrum and other psychotic disorders	29591	9	Unspecified schizophrenia, subchronic
Schizophrenia spectrum and other psychotic disorders	29592	9	Unspecified schizophrenia, chronic
Schizophrenia spectrum and other psychotic disorders	29593	9	Unspecified schizophrenia, subchronic with acute exacerbation
Schizophrenia spectrum and other psychotic disorders	29594	9	Unspecified schizophrenia, chronic with acute exacerbation
Schizophrenia spectrum and other psychotic disorders	29595	9	Unspecified schizophrenia, in remission
Schizophrenia spectrum and other psychotic disorders	F060	10	Psychotic disorder with hallucinations due to known physiological condition
Schizophrenia spectrum and other psychotic disorders	F061	10	Catatonic disorder due to known physiological condition
Schizophrenia spectrum and other psychotic disorders	F200	10	Paranoid schizophrenia
Schizophrenia spectrum and other psychotic disorders	F201	10	Disorganized schizophrenia
Schizophrenia spectrum and other psychotic disorders	F202	10	Catatonic schizophrenia
Schizophrenia spectrum and other psychotic disorders	F203	10	Undifferentiated schizophrenia
Schizophrenia spectrum and other psychotic disorders	F205	10	Residual schizophrenia
Schizophrenia spectrum and other psychotic disorders	F2081	10	Schizophreniform disorder
Schizophrenia spectrum and other psychotic disorders	F2089	10	Other schizophrenia
Schizophrenia spectrum and other psychotic disorders	F209	10	Schizophrenia, unspecified
Schizophrenia spectrum and other psychotic disorders	F22	10	Delusional disorders
Schizophrenia spectrum and other psychotic disorders	F23	10	Brief psychotic disorder
Schizophrenia spectrum and other psychotic disorders	F24	10	Shared psychotic disorder
Schizophrenia spectrum and other psychotic disorders	F250	10	Schizoaffective disorder, bipolar type
Schizophrenia spectrum and other psychotic disorders	F251	10	Schizoaffective disorder, depressive type
Schizophrenia spectrum and other psychotic disorders	F258	10	Other schizoaffective disorders
Schizophrenia spectrum and other psychotic disorders	F259	10	Schizoaffective disorder, unspecified
Schizophrenia spectrum and other psychotic disorders	F29	10	Unspecified psychosis not due to a substance or known physiological condition

## APPENDIX B

**Table:**

Medications
Anesthetics
Propofol
Fentanyl (base) (citrate)
Dexmedetomidine HCl
Rocuronium bromide
Ketamine HCl
Morphine sulfate
Pentobarbital sodium
Etomidate
Vecuronium bromide
Antibiotics
Cephalexin (HCl) (monohydrate)
Antibiotic combinations
Azithromycin (dihydrate)
Ceftriaxone sodium
Sulfamethoxazole and trimethoprim (co‐trimoxazole)
Clindamycin (HCl) (palmitate) (phosphate)
Amoxicillin trihydrate and potassium clavulanate
Doxycycline (calcium) (hyclate) (monohydrate)
Metronidazole (HCl)
Vancomycin (HCl)
Cefazolin sodium
Nitrofurantoin
Ciprofloxacin (HCl)
Penicillin V potassium
Penicillin G benzathine
Antibiotic and steroid combinations
Cefdinir
Ampicillin sodium and sulbactam sodium
Cefotaxime sodium
Penicillin G benzathine and procaine
Ampicillin (anhydrous) (sodium) (trihydrate)
Nafcillin sodium
Cefadroxil (monohydrate)
Cefoxitin sodium
Cefprozil (anhydrous)
Cefixime
Clarithromycin
Tetracycline HCl
Linezolid
Rifaximin
Neomycin sulfate and polymyxin B sulfate
Rifampin
Amoxicillin trihydrate
Minocycline (HCl)
Antidepressants
Sertraline HCl
Fluoxetine HCl
Trazodone HCl
Escitalopram oxalate
Bupropion (HBr) (HCl)
Citalopram HBr
Duloxetine HCl
Venlafaxine
Amitriptyline HCl
Paroxetine (HCl) (mesylate)
Desvenlafaxine
Mirtazapine
Anti‐emetics
Promethazine HCl
Ondansetron HCl
Anti‐epileptics
Valproic acid and derivatives
Clonazepam
Oxcarbazepine
Topiramate
Levetiracetam
Zonisamide
Carbamazepine
Clobazam
Ethosuximide
Lacosamide
Antipsychotics
Risperidone
Olanzapine
Aripiprazole
Haloperidol (decanoate) (lactate)
Quetiapine fumarate
Ziprasidone (HCl) (mesylate)
Chlorpromazine HCl
Clozapine
Asenapine
Paliperidone
Lurasidone HCl
Perphenazine
Anxiolytics
Lorazepam
Diphenhydramine (citrate) (HCl)
Hydroxyzine (HCl) (pamoate)
Midazolam HCl
Alprazolam
Diazepam
Buspirone HCl
*Immune globulin (IVIG)*
Mood stabilizers
Lithium (carbonate) (citrate)
Lamotrigine
Steroids
Dexamethasone
Methylprednisolone
Prednisone
Prednisolone
Hydrocortisone
Beclomethasone dipropionate
Imaging Brain MRI
Head CT
Head ultrasound
**A**bdominal imaging
Abdomen supine X‐ray
Upper abdomen ultrasound real‐time
Noninvasive vascular ultrasound of the abdomen
Retroperitoneum ultrasound real‐time
Abdomen supine upright decubitus and cross‐table X‐ray
Abdomen and pelvis CT scan
Noninvasive vascular Doppler of the abdomen
Lower abdomen MRI
Upper abdomen MRI
Upper abdomen ultrasound B‐mode
Lower abdomen CT scan
Noninvasive vascular ultrasound of thoracic and abdominal aorta
Noninvasive vascular Doppler of thoracic and abdominal aorta
Abdomen arteriography CT scan

Laboratory studies
**A**utoimmune labs
Antinuclear antibody (ANA)
Other specified immunology antibody
Antimicrosomal antibody
Antithyroglobulin antibody
Anti‐DNA antibody
SSA autoantibody
Neuronal antibody
IgG
Anti‐Sm (Smith) antibody
C3
Acetylcholine receptor antibody
SSB autoantibody
Anti GAD 65
C4
Antiribonucleoprotein antibody (RNP antibody)
Anticardiolipin antibody
IgA
Anti‐DNase B antibody
Immunology antibody unspecified
IgG
Rheumatoid factor (RF)
Antineutrophilic cytoplasmic antibody (ANCA) (ACPA)
IgM
Immunoglobulins
Anti‐Scl 70 antibody
Ribosomal P antibody
Total complement activity
Antibody screen
IgG
Other specified immunology test
Immunology test unspecified
Endomysial antibody
Anticentromere antibody
IgE
Neuromyelitis optica
Anti‐smooth muscle antibody
Myelin
Thyroxine binding globulin (TBG)
Insulin antibody
IgD
Immunology profile
Immunology antibody unspecified
Antiadrenal antibody
Anti‐hu TransG
Parietal cell antibody
Reticulin antibody
Intrinsic factor antibody
Chemistry
14‐test chemistry profile
Lipid unspecified
8‐test chemistry profile
Liver profile
Gamma‐glutamyl transpeptidase (GGT)
Alanine aminotransferase (ALT)
Creatinine
Ceruloplasmin
Vitamin B12
Aspartate aminotransferase (AST)
Urea nitrogen
Phosphorus
Magnesium
Alkaline phosphatase
Albumin
Folate
Potassium
Ammonia
Sodium
Total bilirubin
Calcium
Creatine kinase (CK)
Chloride
Carbon dioxide (CO2)
Direct bilirubin
Total protein
Total cholesterol
Copper
Electrolyte profile
Vitamin D
Triglycerides
High‐density lipoprotein (HDL)
Fasting blood glucose
Low‐density lipoprotein (LDL)
Random glucose
Glucose unspecified
Ionized calcium
Kidney profile
Lipase
Lactate dehydrogenase (LD) (LDH)
Chemistry test unspecified
Vitamin B1
Indirect bilirubin
Total protein
Creatinine
Magnesium
Amylase
Prealbumin
Albumin
Homocysteine
Other multitest chemistry profile
Troponin
Calcitriol
Magnesium
Vitamin B6
Tissue transglutaminase (TTG)
Albumin
Alkaline phosphatase
Other specified lipid
Hemoglobin
Bilirubin
Creatinine
Aldosterone
Renin
B‐type natriuretic peptide (BNP)
Cystatin C
Other specified chemistry test
Chemistry test unspecified
Creatine
Creatinine
Phosphorus
Vitamin E
Albumin
8‐test chemistry profile—ionized calcium
Vitamin B3
Liver kidney microsomal antibody
Vitamin A
Ionized calcium
Lipase
CSF studies
Total protein
Glucose unspecified
Aerobic cultures
Specimen preparation
Hematology exam of other body fluid
Other specified immunology antibody
Neuronal antibody
Anti GAD 65
Meningitis/Encephalitis pathogen panel, molecular pathology testing, any method
Other specified amino acids and peptides
Other specified viral antibody
Neopterin
Protein electrophoresis
Herpes virus PCR
Myelin
Other specified catecholamines
Amino acids and peptides unspecified
Polymerase chain reaction (PCR)
Lactic acid
Other specified laboratory test
Vitamin B6
Western equine encephalitis antibody
St Louis equine encephalitis antibody
Enterovirus RNA
Angiotensin‐converting enzyme
Other specified meningitis bacteria
Islet cell antibody (ICA)
Immunoelectrophoresis (IEP)
Enterovirus PCR
Immunology antibody unspecified
Mycoplasma serology
Cryptococcus antigen
Microbial identification nucleic acid probe
West Nile virus PCR
Tetrahydrobiopterin (BH4)
Pyruvic acid
Other specified nonprotein nitrogenous compounds
IgM
Immunoglobulins
Other specified bacteria antibody
Cryptococcus antibody
India ink preparation
Epstein‐Barr virus
Herpes culture
Rubeola antibody
Cytopathology slide reading/interpretation
Cytomegalovirus RT‐PCR
Epstein‐Barr virus PCR
Adenovirus PCR
Varicella PCR
Neuromyelitis optica
Endocrine
Chorionic gonadotropin (HCG)
Random glucose
Insulin
Hemoglobin A1C (HgbA1C)
Chorionic gonadotropin (HCG)
Prolactin
Cortisol
Follicle‐stimulating hormone (FSH)
Luteinizing hormone (LH)
Parathyroid hormone (PTH)
Very long chain fatty acid
Insulin‐like growth factor I (IGF‐I)
Estradiol
Dehydroepiandrosterone (DHEA)
Estrogen
Androsterone
Endocrine – thyroid
Thyroxine (T4)
Free triiodothyronine (free T3)
Triiodothyronine (T3)
Thyroid‐stimulating hormone (TSH)
T3 uptake
TSH receptor antibody
Thyroglobulin
Thyroid profile
Thyroid‐stimulating hormone (TSH)
Free thyroxine
Hematology
Reticulocyte count
Transferrin
Fibrinogen level
Factor VIII‐related antigen
D‐dimer
ABO type antigen
Rh phenotype antigen
Occult blood
Direct antiglobulin test
B cells
Hemoglobin electrophoresis
T cells
Thrombin clotting time (TCT)
Protoporphyrin
Glucose‐6‐phosphate dehydrogenase (G‐6‐PD)
Haptoglobin
Myoglobin
Alpha‐1 antitrypsin
Factor VIII assay
Porphyrin unspecified
White blood cell count with differential
Complete blood count unspecified (CBC)
Hemoglobin
Platelet count
Hematocrit
White blood cell count without differential
Hemoglobin electrophoresis
Factor VII assay
Factor IX assay
Factor VIII‐related antigen
PT and PTT
Protein C
Protein S
Antithrombin III
CBC with differential
CBC without differential
Leukocyte differential count
Lactic acid
Prothrombin time (PT)
Iron
Partial thromboplastin time (PTT)
Ferritin
Uric acid
Peripheral blood smear evaluation
Iron binding capacity (TIBC)
Hematocrit
Hemoglobin S
Infectious disease
Erythrocyte sedimentation rate (ESR)
C‐reactive protein (CRP)
SARS‐CoV‐2 (COVID‐19) molecular pathology testing, any method (DNA probe, PCR, RNA, RT‐PCR)
Gram stain
VDRL
HIV‐1 antibody
Rapid plasma reagin (RPR)
Aerobic cultures
Chlamydia trachomatis, molecular pathology testing, any method
Neisseria gonorrheae, molecular pathology testing, any method
Beta hemolytic streptococcus antigen detection
HIV antigen
SARS‐CoV‐2 (COVID‐19) test, unspecified
Group A Streptococcus, molecular pathology testing, any method
Anti‐streptolysin O titer
Herpes virus PCR
Fluorescent treponemal antibody‐absorption (FTA‐ABS) (FTA)
Lyme disease
Hepatitis C antibody (HCV)
Hepatitis B surface antigen (HBsAg)
Aerobic cultures
Other specified viral antibody
Trichomonas, molecular pathology testing, any method
Aerobic and anaerobic cultures
Strep screen culture
Epstein‐Barr virus
Combo SARS‐CoV‐2 and Multiple Respiratory Viral Organism molecular pathology testing, any method (DNA probe, PCR, RNA, RT‐PCR)
Bacterial test unspecified
Neisseria gonorrheae, molecular pathology testing, any method
Enterovirus PCR
Chlamydia trachomatis, molecular pathology testing, any method
hs‐CRP
Hepatitis test unspecified
Hepatitis B core antibody (HBcAb)
Staphylococcus
Hepatitis B surface antibody (HBsAb)
Respiratory pathogen panel, 12‐25 targets, molecular pathology testing, any method
Mycoplasma serology
HIV‐2 antibody
Hepatitis A viral antibody IgM (HAV‐Ab IgM)
Aerobic cultures
Chlamydia PCR
Varicella PCR
Cytomegalovirus PCR
Other specified meningitis bacteria
West Nile virus antibody
Other specified bacteria antibody
Other specified microbiology test
HIV DNA polymerase chain reaction (PCR)
Epstein‐Barr virus PCR
QuantiFERON TB Gold
Aerobic cultures
Cytomegalovirus antibody
Microbiology test unspecified
Aerobic cultures
Infectious mononucleosis screen
Western equine encephalitis antibody
St Louis equine encephalitis antibody
Mycoplasma pneumoniae, molecular pathology testing, any method
Mycobacteria culture
Hepatitis A viral antibody (HAV) total
Chlamydia pneumoniae, molecular pathology testing, any method
Chlamydia trachomatis/Neisseria gonorrheae combination, molecular pathology testing, any method
Other specified SARS‐CoV‐2 (COVID‐19) test
Molecular pathology unspecified
Neisseria PCR
SARS‐CoV‐2 (COVID‐19) antibody
Other specified viruses
Procalcitonin
Mycoplasma other/unspecified, molecular pathology testing, any method
Group B Streptococcus, molecular pathology testing, any method
Wet prep for parasites
Herpes unspecified
Meningitis viruses
Varicella zoster
Hepatitis B core antibody IgM (HBcAb IgM)
Respiratory pathogen panel, 3–5 targets, molecular pathology testing, any method
Treponema pallidum unspecified
Influenza A and B antigen
Other specified viral culture
Aerobic cultures
Other specified bacteriology
Hepatitis C RNA (HCV RNA)
Chlamydia DNA probe
Lyme PCR
Streptococcus pneumoniae, molecular pathology testing, any method
Treponema pallidum unspecified
VDRL
Meningitis viruses
Microbial identification nucleic acid probe with amplification
Adenovirus PCR
Parechovirus, molecular pathology testing, any method
Enterovirus RT‐PCR
Bordetella pertussis PCR
Neisseria gonorrhoeae
Herpes simplex antibody
West Nile virus antibody
Influenza virus PCR
Escherichia coli PCR
Cryptococcus, molecular pathology testing, any method
Neisseria meningitidis, molecular pathology testing, any method
Listeria, molecular pathology testing, any method
Haemophilus influenzae, molecular pathology testing, any method
Mycoplasma pneumoniae, molecular pathology testing, any method
Methicillin‐resistant staphylococcus (MRSA) PCR
Aerobic cultures
Aerobic cultures
Brucella species
Rickettsial antibody
H influenza
Cryptosporidium
Respiratory pathogen panel, unspecified # targets, molecular pathology testing, any method
Toxoplasma serology
Cytomegalovirus DNA probe
Epstein‐Barr virus RT‐PCR
Herpes virus DNA probe
Chlamydia PCR
Chlamydia trachomatis/Neisseria gonorrheae combination, molecular pathology testing, any method
Anaerobic cultures
Bacteria antibody unspecified
Corynebacterium diphtheriae
Streptococcus unspecified
Streptococcus pneumoniae
Other specified parasitic antibody
Malarial smear
Adenovirus
Respiratory syncytial virus antigen
HIV‐1 RNA
Hepatitis B core antibody (HBcAb)
Hepatitis Be antibody (HBeAb)
Hepatitis Be antigen (HBeAg)
Hepatitis B surface antigen (HBsAg)
Clostridium difficlie PCR
Chlamydia antibody
Cryptococcus antigen
Fungal culture
Viral antibody unspecified
Meningitis viruses
Rubella antibody
Influenza A and B antibody
Coxsackievirus group A antibody
Coxsackievirus group B antibody
Echovirus test
Parvovirus test
HIV western blot
Other specified hepatitis test
SARS‐CoV‐2 (COVID‐19) antigen
Other specified viral culture
Epstein‐Barr virus PCR
Herpes virus PCR
Varicella PCR
Legionella pneumophila antigen
Staphylococcus
H influenza
Leptospira antibody
Yersinia culture
Epstein‐Barr virus
Herpes unspecified
Herpes simplex virus antigen
Parainfluenza antigen
Metapneumovirus (hMPV)
Poliovirus test
Respiratory syncytial virus antigen
Respiratory syncytial virus culture
HIV antigen neutralization
Hepatitis B viral DNA
Other specified viral culture
Other specified viruses
Common human coronavirus (229E, NL63, OC43, HKU1, etc.), molecular pathology testing
Cytomegalovirus PCR
Cytomegalovirus DNA probe
Epstein‐Barr virus DNA probe
Herpes simplex/varicella zoster virus combination, molecular pathology testing
West Nile virus RT‐PCR
Parainfluenza virus PCR
Parvovirus PCR
Parvovirus PCR
Respiratory syncytial virus RT‐PCR
Other specified viral molecular pathology
Shiga/Shiga‐like toxin, molecular pathology testing
Chlamydia RNA
Neisseria DNA probe
Varicella PCR
BK virus PCR
Chlamydia pneumoniae, molecular pathology testing
Toxicology
Therapeutic drug monitoring (TDM) unspecified
Therapeutic drug monitoring (TDM) unspecified
Lidocaine therapeutic drug monitoring
Amitriptyline therapeutic drug monitoring
Alcohol toxicology unspecified
Toxicology unspecified
Toxicology unspecified
Benzodiazepine toxicology
Cannabinoid toxicology
Amphetamine toxicology
Acetaminophen toxicology
Barbiturate toxicology unspecified
Morphine and derivatives toxicology
Cocaine toxicology
Ethanol toxicology
Drug monitoring other therapeutic category
Phencyclidine toxicology
Other specified toxicology
Salicylate toxicology
Meprobamate therapeutic drug monitoring
Cannabinoid toxicology
Valproic acid therapeutic drug monitoring
Lead toxicology
Lithium therapeutic drug monitoring
Morphine and derivatives toxicology
Cocaine toxicology
Amphetamine toxicology
Barbiturate toxicology unspecified
Lead toxicology
Toxicology unspecified
Phencyclidine toxicology
Ethanol toxicology
Benzodiazepine toxicology
Mercury toxicology
Salicylate toxicology
Arsenic toxicology
Other antidepressant/antipsychotic/antianxiety therapeutic drug monitoring
Arsenic toxicology
Arsenic toxicology
Mercury toxicology
Lead toxicology
Mercury toxicology
Ethanol toxicology
Drug monitoring other therapeutic category
Heavy metal toxicology unspecified
Porphyrin unspecified
Dilute Russell's viper venom time (DRVVT)
Acetaminophen toxicology
Other antidepressant/antipsychotic/antianxiety therapeutic drug monitoring
Theophylline therapeutic drug monitoring
Zinc toxicology
Phenobarbital therapeutic drug monitoring
Cadmium toxicology
Other specified therapeutic drug monitoring
Sedatives and hypnotics therapeutic drug monitoring
Cadmium toxicology
Keppra therapeutic drug monitoring
Narcotic other than morphine toxicology
Trileptal therapeutic drug monitoring
Zinc toxicology
Carbamazepine therapeutic drug monitoring
Antidepressant/antipsychotic/antianxiety therapeutic drug monitoring unsp
Cadmium toxicology
Carbon monoxide
Anticonvulsant therapeutic drug monitoring unspecified
Other specified toxicology
Heavy metal toxicology unspecified
Arsenic toxicology
Lead toxicology
Mercury toxicology
Other specified toxicology
Anticonvulsant therapeutic drug monitoring unspecified
Phenytoin therapeutic drug monitoring
Other specified anticonvulsant therapeutic drug monitoring
Methotrexate therapeutic drug monitoring
Toxicology unspecified
Heavy metal toxicology unspecified
Manganese toxicology
Other specified alcohol toxicology
Phencyclidine toxicology
Cannabinoid toxicology
Acetaminophen toxicology
Urine studies
Chemical examination of urine
Copper
Porphobilinogen
Amino acids and peptides unspecified
Total protein
Porphyrin unspecified
Methylmalonic acid
Albumin
Cadmium toxicology
Microscopic examination of urine
Sodium
Osmolality
Metanephrines
Creatinine
Porphobilinogen
Potassium
Total protein
Urinalysis unspecified
Chloride
Creatine
Specimen collection
Metabolic screen
Carnitine
Glucose unspecified
Urea nitrogen
Uric acid
Microalbumin
Complete urinalysis
Macroscopic urinalysis
Copper

Other clinical resources
Electroencephalogram
Electrocardiogram
Therapies (PT/OT/ST)
